# Human cytomegalovirus infection and its association with gestational diabetes mellitus during pregnancy

**DOI:** 10.7717/peerj.12934

**Published:** 2022-02-15

**Authors:** Yunyang Wang, Xianjuan Zhang, Xu Zheng, Guanghui Song, Lina Fang, Yangang Wang, Bin Wang

**Affiliations:** 1 Department of Endocrinology and Metabolism, The Affiliated Hospital of Qingdao University, Qingdao, China; 2 School of Basic Medicine, Qingdao University, Qingdao, China; 3 Department of Laboratory Medicine, Qingdao Municipal Hospital, Qingdao, China; 4 Department of Clinical Laboratory, The Affiliated Hospital of Qingdao University, Qingdao, China; 5 Department of Laboratory Medicine, Zibo Maternal and Child Health Hospital, Zibo, China

**Keywords:** Gestational diabetes mellitus, Human cytomegalovirus infection during pregnancy, Inflammatory factor, HCMV antibody IgM

## Abstract

**Background:**

Infection is an important risk factor for gestational diabetes mellitus (GDM), while infection of human cytomegalovirus (HCMV) with GDM remains unclear and rarely reported. This study aimed to investigate the association of HCMV infection and serum inflammatory factor levels in pregnancy with GDM.

**Methods:**

This prospective study included pregnant women who attended at Affiliated Hospital of Qingdao Hospital and Zibo Maternal and Child Health Hospital between December 2018 and August 2020. HCMV specific IgM and serum levels of inflammatory factors, including TNF-α, IL-6, and IL-1β, were analyzed.

**Results:**

A total of 5,316 pregnant women were included (415 with GDM (107 with HCMV+GDM+ and 308 with HCMV-GDM+) and 4901 GDM-free (759 with HCMV+GDM- and 4142 with HCMV-GDM-)). The prevalence of GDM was 7.81%. The rate of activation of HCMV was 16.29%. Specifically, 107 and 759 women in the GDM and control group exhibited HCMV infection, with positive rates of25.78% and 15.48%, respectively (*P* < 0.01). TNF-α, IL-6, and IL-1β at 24–28 weeks of gestation were significantly higher in women with GDM and HCMV infection than inthe other groups (all *P* < 0.01). Multivariable analysis showed that HCMV positive (OR = 1.851; 95% CI [1.425–2.403]; *P* < 0.001), IL-6 (OR = 1.010; 95% CI [1.002–1.018]; *P* = 0.013), and IL-1β (OR = 1.410; 95% CI [1.348–1.474]; *P* < 0.001) were all significantly correlated with GDM.

**Conclusion:**

This study suggests HCMV infection during pregnancy is an independent risk factor of GDM and could significantly increase its incidence. Further studies are needed to elucidate possible mechanisms underlying associations between HCMV infection and GDM.

## Introduction

Human cytomegalovirus (HCMV) is a β herpesviridae subfamily, a double-stranded DNA virus. The infection rate of HCMV, which has species specificity, is 65%–80% in developing countries and 80%–100% in developed countries (Onno et al., 2000). HCMV has co-existed with humans for eons and has become the most widespread infection globally. Life-long dormant infection can exist after initial infection with HCMV. The humoral immunity and cellular immunity established play important roles in the occurrence, progression, and outcomes of HCMV infection. Antibodies can restrict the replication of HCMV, thus providing a certain resistance against seconder-infection of the same viral strain; however, antibodies may not prevent the activation of or re-infection with HCMV (Griffiths, Baraniak & Reeves, 2015). The specific cellular immunity generated after the initial infection can eliminate the viremia and promote latency of the virus, which may persist for the lifetime of the host (Simpson et al., 2016). HCMV infection or re-emerging infection during pregnancy is very common. Several previous studies have reported that HCMV infection can directly influence pregnancy outcomes, including pre-term birth, low birth weight, megaloblastic anemia, antepartum hemorrhage, deformity, and gestational hypertension. However, very few studies have delved into the influences of the infectious state of HCMV during pregnancy on the development of gestational diabetes mellitus (GDM).

GDM refers to the different degrees of impaired glucose tolerance that occur or are initially discovered during pregnancy, accounting for 80%–90% of all GDM associated with pregnancy. GDM is commonly found during pregnancy and often severely influences maternal and fetal health (Cho et al., 2018). In recent years, the global incidence of GDM has increased. In China, the reported incidence of GDM is 4.3%–5.1%, which is gradually approaching the incidence described in developed countries in Europe and the United States of America (5%–8%). Blood glucose levels in about 75% of GDM patients return to normal after delivery. However, GDM in about 6%–10% of the patients will progress to type 2 diabetes mellitus within 2–10 years, and 50% of the GDM patients will be patients with type 2 diabetes mellitus throughout their lives (Feig et al., 2018). In 1979, the WHO classified GDM as an independent type of diabetes mellitus though the exact molecular mechanisms involved in its pathogeneses are still not fully understood. (Thomas & Philipson, 2015). GDM is caused by the inhibition of insulin production triggered by the synergistic effect of multiple factors, partly due to the secretion of inflammatory cytokines and the regulation of pro-inflammatory signaling pathways. TNF-α, IL-6, IL-1β, C-reactive protein, and NF-κB are highly probable biomarkers of GDM and may eventually be utilized for GDM screening in early and late pregnancy (Khambule & George, 2019).

In China, most individuals have been infected with HCMV prior to18 years of age, and thus the HCMV infections during pregnancy are mainly due to activation. The immunological memory induced by infection during pregnancy may generate chronic inflammation and disturbance, and the processes could interact with maternal immunity to trigger the occurrence and progression of GDM. Alternatively, GDM is also a chronic inflammation, which is thus induced by HCMV infection and may participate in the development of GDM occurrence and progression. Nonetheless, the exact association between HCMV infection during pregnancy and GDM requires further scrutiny. Several retrospective studies have suggested that hepatitis B virus (HBV) surface antigen (HBsAg)-positive women are at higher risk of GDM (Lao et al., 2007; Peng et al., 2019; Tan et al., 2016). Other studies on the mechanisms underlying the effects of HBV infection on GDM have shown that HBV infection could aggravate the chronic inflammation state related to insulin resistance in pregnant women (Fernández-Real et al., 1998; Gomes et al., 2013; Kirwan et al., 2002; Sheron et al., 1991). However, no previous studies have studied the mechanisms underlying the influences of HCMV infection on GDM yet.

Therefore, our study aimed to examine the association between activation of HCMV during pregnancy and GDM incidence and the association between the changes of inflammatory factors induced by HCMV infection and GDM incidence.

## Methods

### Study design and subjects

This prospective study included pregnant women receiving prenatal care in the Zibo Maternal and Child Health Hospital and Affiliated Hospital of Qingdao Hospital between December 2018 and August 2020. This study was approved by the Ethics Committee of the Zibo Maternal and Child Health Hospital. The criteria for inclusion were pregnant women at 24–28 weeks, no history of diabetes mellitus prior to pregnancy, complete prenatal clinical examination record tables available, and no other endocrine diseases. The exclusion criteria included recent medication usage, which could influence blood glucose levels.

The women were divided into the GDM positive group and GDM negative group, according to the blood glucose examination results.

### Data collection

Data were collected from the electronic medical records database of the hospital. Demographic data, including age, body-mass index (BMI), and marital status, as well as clinical data, including a history of cesarean section, gravidity, HCMV infection status, and serum levels of inflammatory factors (TNF-α, IL-6, and IL-1β), were collected.

### HCMV-IgM ELISA

Fasting venous blood (5 ml) was collected prior to 9 am at 24–28 weeks and was centrifuged at 2000–3000 rpm for 20 min, and serum was collected for assay of HCMV-IgM and HBV. The qualitative detection kit for HCMV-IgM (INS1010102; Inselisa Biotechnology Co., Ltd, Huangshi, China) was used to evaluate the infection of HCMV-IgM by ELISA method, using the automatic enzyme immunoassay analyzer (BIOBASE1001, Beisheng Medical Equipment Co., LTD, Jinan, China), and positivity of HCMV-IgM was construed as the activation of HCMV-IgM. Additionally, 1–1.5 ml middle segment urine was collected into a centrifugation tube, centrifuged at 12,000 rpm for 10 min, and the supernatant discarded. The HCMV-DNA in urine was detected *via* the HCMV nucleotide acid detection kit (4926-2011; Acon Biotechnology Co., Ltd) by fluorescent quantitative PCR, using the fluorescent quantitative PCR analyzer (LightCycler 480 II; Roche Diagnostics).

### Inflammatory markers

The ELISA kit utilized for TNF-α was E-EL-H0109C (Elabscience Biotechnology Co., Ltd, Wuhan, China), IL-6 kit was SBJ-H0465 (SenBeiJia Biotechnology Co., Ltd, Nanjing, China), and human IL-1β kit was 583311-96 (AmyJet Scientific Inc, Wuhan, China).

### Grouping

According to the status of HCMV infection, the women were divided into the GDM positive group and GDM negative group as follows: GDM positive group: GDM+/HCMV+ (G+/H+): HCMV activation; GDM+/HCMV − (G+/H−): HCMV not activated. GDM negative group: GDM −/HCMV+(G−/H+): HCMV activation; GDM −/HCMV − (G−/H−): HCMV not activated.

### Diagnostic criteria of GDM

GDM was diagnosed according to the standards of the American Diabetes Association (American Diabetes Association, 2018). In brief, an oral glucose tolerance test (OGTT) was performed. Patients who met one or more of the following criteria were diagnosed with GDM: (1) fasting OGTT ≥5.1 mmol/L; (2) OGTT ≥10.0 mmol/L at 1 h after glucose intake; (3) OGTT ≥8.5 mmol/L at 2 h after glucose intake.

### Statistical analysis

Normality testing was performed for all the quantitative data first. Normally distributed quantitative data were described as Mean ± SD and compared by independent *t*-test for differences between two groups. Quantitative data found not to be normally distributed were described as median (min-max) and compared by Mann–Whitney U test for differences between two groups. Qualitative data were described by frequencies and percentages n (%) and compared by chi-square test for differences. Univariable and multivariable logistic regression was performed, using GDM as the dependent factor. In the multivariable logistic regression, if the independent factors were associated with each other, the factors were included in different models of multivariable logistic regressions. R 3.6.1 software (2019) was used for the statistical analysis in this study.

## Results

### Demographic characteristics associated with GDM

5316 pregnant women were included in this study. Ages ranged from 23 to 44 years, and gestational weeks ranged from 24 to 28 weeks.415 were diagnosed with GDM, the prevalence of which was 7.81%. HCMV-IgM, the indicator of HCMV activation, was measured in blood samples of the 5216 pregnant women at 24–28 weeks of gestation, and the results showed that HCMV activation positivity in 866 women, with an overall positive rate of 16.29%. Specifically, 107 and 759 women in the GDM positive group and GDM negative group were exhibited infection with a positive rate of 25.78% and 15.49%, respectively (*P* < 0.01).

Basic demographic and clinical characteristics are depicted in Table 1. The mean age of women in the GDM positive group (*n* = 415) was significantly higher than those in GDM negative group (*n* = 4901) (33.34 ± 3.61 *vs.* 28.91 ± 4.43 years, *P* < 0.001), and the BMI was significantly higher in GDM positive group than GDM negative group (*P* < 0.001). The incidence of GDM was significantly higher in the 2nd parity than the 1st parity (*P* < 0.001). In contrast, marital status and history of a cesarean section did not significantly influence the incidence of GDM (*P* > 0.05) (Table 1). Fasting blood glucose and insulin levels were higher in the GDM group (both *P* < 0.001).

**Table 1:** Baseline characteristics.

Characteristics	GDM (+) (*n* = 415)	GDM (−) (*n* = 4901)	*P* value
Age, years	33.34 ± 3.61	28.91 ± 4.43	<0.001
BMI	27.59 ± 4.16	24.60 ± 3.94	<0.001
Marital status			>0.05
Married	413 (99.52)	4879 (99.55)	
Unmarried	2 (0.48)	22 (0.45)	
History of cesarean section	115 (27.71)	1379 (28.14)	>0.05
Parity			<0.001
1	149 (35.90)	2744 (55.99)	
2	266 (64.10)	2157 (44.01)	
Serum inflammatory factor levels			
TNF-α	5.03 ± 2.82	4.66 ± 2.30	0.009
IL-6	130.24 ± 39.18	113.15 ± 39.38	<0.001
IL-1β	121.51 ± 9.89	83.14 ± 11.15	<0.001
Fasting blood glucose, mmol/L	5.35 ± 0.66	4.57 ± 0.26	<0.001
Fasting insulin, mIU/L	10.22 ± 3.02	8.29 ± 2.92	<0.001

### Association of HCMV infection and GDM with inflammatory factors

Inflammatory factors levels, including TNF-α, IL-6, and IL-1β, were measured from blood samples at 24–28 weeks of gestation. These levels were significantly higher in the GDM positive group than the GDM negative group (all *P* < 0.01; Table 1).

The cohort of pregnant women was divided into four groups according to HCMV infection and GDM status as follows: HCMV+GDM+ group, HCMV+GDM- group, HCMV-GDM+ group, and HCMV-GDM-. The TNF-α (5.86 ± 3.23 *vs* 4.74 ± 2.61, *P* = 0.009), IL-6(142.45 ± 39.25 *vs* 125.99 ± 38.32, *P* = 0.001), and IL-1β (124.73 ± 10.57 *vs* 120.39 ± 9.41, *P* = 0.001)levels were significantly higher in the HCMV+GDM+ group than the HCMV-GDM+ group (Table 2).The TNF-α (4.96 ± 2.42 *vs* 4.60 ± 2.28, *P* = 0.001), IL-6 (119.81 ± 41.20 *vs* 111.93 ± 38.92, *P*<0.001), and IL-1β (84.71 ± 11.72 *vs* 82.86 ± 11.02, *P* < 0.001) levels were also significantly higher in the HCMV+GDM- group than the HCMV-GDM- group (Table 2).

**Table 2:** Association between HCMV infection and inflammatory factors.

Levels of inflammatory factors	GDM (+)		*P* value	GDM (−)		*P* value
	G+/H+ (*n* = 107)	G+/H− (*n* = 308)		G−/H+ (*n* = 759)	G−/H− (*n* = 4142)	
TNF-α	5.86 ± 3.23	4.74 ± 2.61	0.009	4.96 ± 2.42	4.60 ± 2.28	0.001
IL-6	142.45 ± 39.25	125.99 ± 38.32	0.001	119.81 ± 41.20	111.93 ± 38.92	<0.001
IL-1β	124.73 ± 10.57	120.39 ± 9.41	0.001	84.71 ± 11.72	82.86 ± 11.02	<0.001

**Notes.**

G+/H+, GDM positive, HCMV activation infection; G+/H −, GDM positive, HCMV not activated; G −/H+, GDM negative, HCMV activation infection; G −/H −, GDM negative, HCMV not activated.

### Multivariable analysis using GDM as the dependent factor

Considering that HCMV has a strong correlation with inflammatory factors, we carried out logistic multivariable analysis included age, BMI, parity, and HCMV positive. We also carried out logistic multivariable analysis included age, BMI, parity, TNF-α, IL-6, and IL-1β. Multivariable analysis showed that HCMV positive (OR = 1.851; 95% CI = 1.425 –2.403; *P* < 0.001), IL-6 (OR = 1.010; 95% CI = 1.002 –1.018; *P* = 0.013), and IL-1β (OR = 1.410; 95% CI = 1.348 –1.474; *P* < 0.001) were all significantly correlated with GDM (Table 3).

**Table 3:** Multivariate analysis with GDM as the dependent factor.

Variables	Univariate analysis		Multivariate analysis 1#		Multivariate analysis 2#	
	OR (95% CI)	*P* value	OR (95% CI)	*P* value	OR (95% CI)	*P* value
Age	1.274 (1.241–1.308)	<0.001	1.279 (1.244–1.316)	<0.001	1.323 (1.223–1.430)	<0.001
BMI	1.213 (1.181–1.247)	<0.001	1.212 (1.177–1.247)	<0.001	1.252 (1.160–1.351)	<0.001
Parity						
1	Reference	/	Reference	/	Reference	/
2	2.271 (1.844–2.797)	<0.001	2.306 (1.839–2.891)	<0.001	4.256 (2.344–7.725)	<0.001
HCMV positive	1.896 (1.502–2.394)	<0.001	1.851 (1.425–2.403)	<0.001		
TNF-α	1.069 (1.025–1.115)	0.002			0.997 (0.886–1.123)	0.964
IL-6	1.011 (1.008–1.014)	<0.001			1.010 (1.002–1.018)	0.013
IL-1β	1.399 (1.348–1.452)	<0.001			1.410 (1.348–1.474)	<0.001

**Notes.**

1#: The independent factors include age, BMI, parity, and HCMV positive.

2#: The independent factors include age, BMI, parity, TNF-α, IL-6, and IL-1β.

## Discussion

This study aimed to explore the association of HCMV infection and serum inflammatory factor levels in pregnant women with GDM. The findings suggest that maternal HCMV activation during pregnancy could independently influence GDMand also demonstrated that the pregnant women with active HCMV were at a higher risk of developing GDM. It is the first report of such findings to date to the best of our knowledge. The analysis of demographic characteristics showed that older age and higher body weight could be independent risk factors associated with higher GDM incidence, which is in agreement with previous findings (Challis et al., 2009). Additionally, thisstudy showed that parity was associated with the incidence of GDM (the prevalence of GDM was significantly higher in women of the second parity than at first), suggesting that it could be associated with the relatively older ages of the women.

Previous studies have demonstrated that GDM is associated with inflammation during pregnancy (Peng et al., 2019). Regarding the influence of viral infection during pregnancy, previous studies demonstrated that HCMV infection could directly influence pregnancy outcomes such as pre-term birth, low birth weight, megaloblastic anemia, antepartum hemorrhage, deformity, and gestational hypertension (Sánchez-Luquez et al., 2021; Yamada et al., 2018). In addition, HBV infection has been reported to be positively correlated with the risk of GDM (Peng et al., 2019; Tan et al., 2016). The differences in the association of viral infection during pregnancy with certain gestational diseases, such as GDM, could possibly be based on the difference in the ethnicities of these women. Furthermore, the prevalence of HCMV infection in women of different ethnicities and ages, as well as the genetic backgrounds of the patients, were also different (Fowler et al., 2018; Lee et al., 2020). These factors could influence the evaluation of the association between HCMV infection during pregnancy and GDM risk.

**Figure 1:**
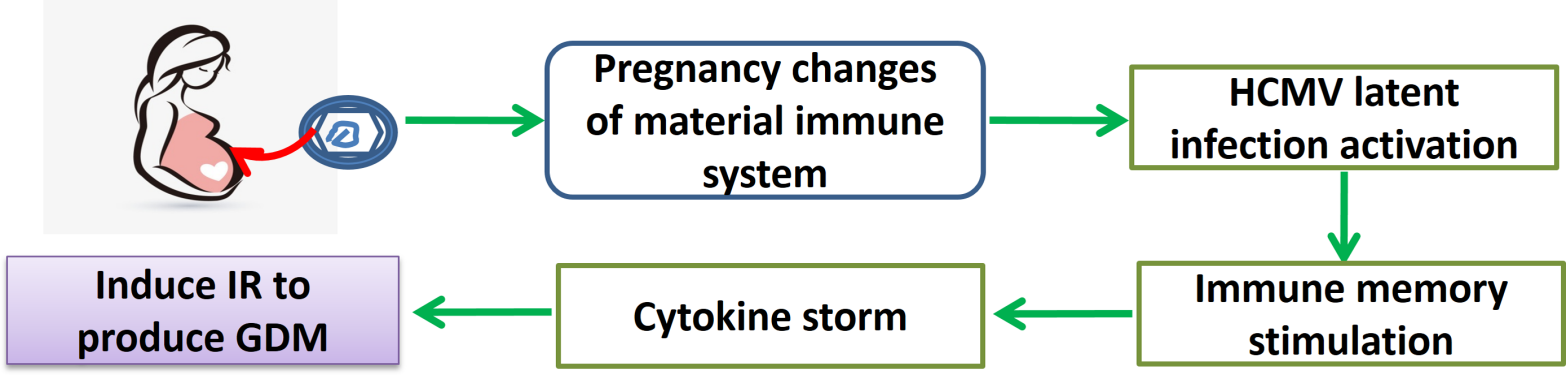
Schematic representation of the possible mechanism for the association between HCMV and GDM, with particular attention to inflammatory markers.

Figure 1 presents a schematic of the possible factors linking HCMV activation and GDM, but the exact mechanisms underlying the association between HCMV infection during pregnancy and GDM remain unclear. Still, HCMV activation is associated with elevated inflammation (Garcia-Torre et al., 2021; Mundkur et al., 2012; Simanek et al., 2011), and previous studies showed that the pathogenesis of GDM involves inflammation, either acute or chronic, that leads to insulin resistance (Gomes et al., 2013; McElwain, McCarthy & McCarthy, 2021; Pantham, Aye & Powell, 2015). In addition to the effects of pregnancy itself, the factors involved in inflammatory status include the elevation of anti-inflammatory factors, such as IL-2, IL-6, IL-10, and tumor necrosis factor-alpha (TNF-α), which could be induced by the activation of HCMV during pregnancy (Chudnovets et al., 2020; McElwain, McCarthy & McCarthy, 2021). In addition, a persistent HCMV infection induced by the unique immune status during pregnancy could also be the cause of insulin resistance and glucose intolerance (McElwain, McCarthy & McCarthy, 2021).

To the best of the authors’ knowledge, no previous data are available regarding the influence of HCMV infection on inflammation during pregnancy. Research on the mechanisms underlying the influence of HBV infection on GDM showed that HBV infection during pregnancy could worsen the chronic inflammatory state and contribute to GDM (Gomes et al., 2013). Our study investigated the association of HCMV antibody types and GDM, which showed statistically significant associations. In addition, we evaluated the association between HCMV activation (HCMV-IgM+) during pregnancy and GDM, which showed that the rate of activation of HCMV was 25.78% in the GDM positive group, which was significantly higher than the 15.48% in the GDM negative group (*P* < 0.001). These findings suggest for the first time an association between the activation of HCMV (HCMV-IgM+) during pregnancy and GDM incidence.

In order to further investigate the mechanisms involved in the induction of GDM by HCMV activation during pregnancy, the blood samples from our study subjects were analyzed for inflammatory factors. The findings showed that the TNF-α, IL-6, and IL-1β levels in the women with GDM and HCMV activation were significantly higher than in the other groups (*i.e.,* GDM without HCMV activation, no GDM but with HCMV activation, and no GDM and no HCMV activation). Previous studies have demonstrated that inflammation during pregnancy is a cause of GDM (American Diabetes Association, 2018; Khambule & George, 2019; McElwain, McCarthy & McCarthy, 2021; Pantham, Aye & Powell, 2015). The findings of the present study support the assertion that the inflammation induced by HCMV activation during pregnancy could be a cause of GDM, but the detailed pathogenesis still needs to be investigated.

There are several limitations to this study. First, this only examined active infection of HCMV during pregnancy, which was positively correlated with GDM as the serum HCMV-IgM detection. However, the power of deducing an actual causal relationship on GDM in this study is limited. Therefore, large-scale prospective studies are needed to verify any possible causal relationships. Second, since there is no animal model of HCMV infection, the results of this study cannot be replicated in animals, and the exact mechanisms cannot be determined. Third, several studies examined the pathogenesis of GDM. Infection during pregnancy has traditionally been thought to be associated with GDM. In addition, an individual’s genetic factors are associated with the onset of GDM. This study involved only examined HCMV active infection during pregnancy. The clinical morbidity associated with GDM and the molecular mechanisms of GDM is only speculative since there is a lack of studies. Fourth, this study cannot confirm the relationship between HCMV-activated infection in pregnancy and GDM. Fifth, the relationship between genetic susceptibility and HCMV infection and GDM must be confirmed. Future studies will examine large cohorts of patients and include immunology, virology, and genetic data to examine individual genetic susceptibility, inflammation, and the immune response to HCMV active infection and the development of GDM. The results should lay a research foundation for the future establishment of new detection methods to evaluate the risk of GDM with HCMV active infection during pregnancy and, at the same time, establish a scientific basis for the intervention, treatment, and prevention of GDM.

## Conclusion

Using the large number of controlled clinical cases, this study observed that the occurrence or development of GDM is associated with HCMV activation during pregnancy. The development of GDM and type 2 diabetes involves a chronic inflammatory state exacerbated by HCMV activation. The present study suggested that GDM is associated with inflammation and HCMV activation, but the exact causal relationship could not be established due to the study design. HCMV has multiple open reading frameworks during infection and replication. Its chronic persistent infection, reactivation infection, and recovery involve complex immune responses, and the protein molecules encoded by the virus are numerous. The genetic background of infected persons and the possible cross-reaction of autoimmune molecules in some infected persons might be related to the occurrence or development of GDM. Further molecular mechanism studies are expected to reveal the existence of the correlation, which is of great significance for the prevention, treatment, and prognosis monitoring of GDM.

##  Supplemental Information

10.7717/peerj.12934/supp-1 Supplemental Information 1 Raw data

## Additional Information and Declarations

### Data Availability

The following information was supplied regarding data availability: The raw data is available in the Supplemental File.
